# The challenges for developing prognostic prediction models for acute kidney injury in hospitalized children: A systematic review

**DOI:** 10.1002/ped4.12458

**Published:** 2024-12-11

**Authors:** Chen Wang, Xiaohang Liu, Chao Zhang, Ruohua Yan, Yuchuan Li, Xiaoxia Peng

**Affiliations:** ^1^ Center for Clinical Epidemiology and Evidence‐based Medicine Beijing Children's Hospital, Capital Medical University, National Center for Children's Health Beijing China; ^2^ Outpatient Department Beijing Children's Hospital, Capital Medical University, National Center for Children's Health Beijing China

**Keywords:** Acute kidney injury, Hospitalized children, Prognostic prediction models

## Abstract

**Importance:**

Acute kidney injury (AKI) is common in hospitalized children which could rapidly progress into chronic kidney disease if not timely diagnosed. Prognostic prediction models for AKI were established to identify AKI early and improve children's prognosis.

**Objective:**

To appraise prognostic prediction models for pediatric AKI.

**Methods:**

Four English and four Chinese databases were systematically searched from January 1, 2010, to June 6, 2022. Articles describing prognostic prediction models for pediatric AKI were included. The data extraction was based on the CHecklist for critical Appraisal and data extraction for systematic Reviews of prediction Modelling Studies checklist. The risk of bias (ROB) was assessed according to the Prediction model Risk of Bias Assessment Tool guideline. The quantitative synthesis of the models was not performed due to the lack of methods regarding the meta‐analysis of prediction models.

**Results:**

Eight studies with 16 models were included. There were significant deficiencies in reporting and all models were considered at high ROB. The area under the receiver operating characteristic curve to predict AKI ranged from 0.69 to 0.95. However, only about one‐third of models have completed internal or external validation. The calibration was provided only in four models. Three models allowed easy bedside calculation or electronic automation, and two models were evaluated for their impacts on clinical practice.

**Interpretation:**

Besides the modeling algorithm, the challenges for developing prediction models for pediatric AKI reflected by the reporting deficiencies included ways of handling baseline serum creatinine and age‐dependent blood biochemical indexes. Moreover, few prediction models for pediatric AKI were performed for external validation, let alone the transformation in clinical practice. Further investigation should focus on the combination of prediction models and electronic automatic alerts.

## Graphical abstract

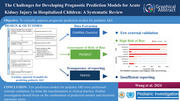

## INTRODUCTION

Acute kidney injury (AKI) is a clinical syndrome associated with a rapid decline of renal function in a short period; it is common in hospitalized children.^1^, ^2^ Previous studies in the United States and the United Kingdom both show that the incidence of AKI in community and hospitalized children is about 1/1000 person‐year and 10% respectively.^3^, ^4^, ^5^ A Chinese study reported an incidence of AKI of 10.2% involving 3 044 224 patients under the age of 18 years admitted to 25 Chinese regional medical centers.^6^, ^7^ The concern is that AKI not only rapidly progresses into chronic kidney disease, even to the extent of requiring renal replacement therapy (RRT) if not diagnosed in a timely manner,^8^ but is also independently associated with higher mortality and a longer admission time of hospitalized children.^2^, ^3^, ^9^ The prospective cohort study in Wales showed that 15.3% of children with repeated episodes of AKI had higher 30‐day mortality (11.6% *vs*. 4.6%) and higher residual renal impairment (13.3% *vs*. 5.4%) compared with children experiencing only one AKI episode.^3^ In a prospective multicenter and international cohort study of 4984 patients admitted to pediatric intensive care units between 3 months and 25 years of age, the mortality in patients with AKI stages 2 or 3 was significantly higher than that in patients who did not develop AKI (11% *vs*. 3.4%).^2^, ^10^

The challenge revolving around AKI is that the onset and progression of AKI are usually imperceptible and its diagnosis is mainly based on serum creatinine (SCr) and urine output.^11^, ^12^ For example, according to the modified KDIGO criterion, pediatric AKI is diagnosed when the SCr rises to 0.5 mg/dL or more within 48 h, or rises by a multiple of 1.5 or more of the baseline SCr within 7 days.^13^, ^14^ However, a continuum of injury exists long before sufficient loss of excretory kidney function can be measured with SCr. Due to these limitations, researchers are committed to developing AKI prediction models in order to identify AKI early, limiting the potential risk of AKI and improving children's prognosis.^15^ Although it is feasible to develop and validate AKI prediction models for children,^16^ the clinical applicability of existing models needs to be further investigated for several reasons: 1) With potential predictors and models of AKI continuously increasing, the results of new studies are inconsistent and have presented contradictory findings.^17^ For example, it is controversial whether the baseline SCr should be used as a predictor for predicting AKI when AKI was diagnosed according to the change of SCr;^18^ 2) Study conclusions are potentially unreliable due to substandard reporting of methodology and results;^19^ 3) The population used to train the model is not well representative, resulting in poor extrapolation of the model; 4) The prediction models have been rarely transformed into clinical automatic warning systems, and the clinical applicability of the existing models remains to be evaluated.^15^, ^20^ Moreover, some results have demonstrated the differences of blood biochemical reference intervals (RIs) between age.^21^, ^22^ Thus, the challenge in developing predictive models for children is how to reasonably manage the predictive factors in accordance with the trends of age change.

Following relevant guidelines, including the CHecklist for critical Appraisal and data extraction for systematic Reviews of prediction Modelling Studies (CHARMS),^23^ the Prediction model Risk of Bias Assessment Tool (PROBAST)^24^ and Transparent Reporting of a multivariable prediction model for Individual Prognosis or Diagnosis (TRIPOD),^25^ this review aims to systematically appraise prognostic prediction models for AKI in hospitalized children, focusing on the risk of bias (ROB) and the clinical applicability of the established prediction models in children so that limitations in methodology for establishing prediction models of pediatric AKI could be summarized, which could provide a recommendation for the subsequent development and applicability of prediction models for AKI in hospitalized children.

## METHODS

### Registration

This systematic review named “Prognosis Prediction Models for Acute Kidney Injury in Children: A Systematic Review” was registered on the PROSPERO platform with registration number “CRD42022353117” (https://www.crd.york.ac.uk/PROSPERO/display_record.php?RecordID=353117).

This review was reported according to the Preferred Reporting Items for Systematic Review and Meta‐Analysis (PRISMA) statement.^26^ Due to the formal methods for meta‐analysis of prediction models not yet being fully developed,^27^ the quantitative synthesis of the models was not performed.

### Information sources, search strategy, and eligibility criteria

Search terms included controlled vocabulary and keywords, acute kidney injury or acute renal failure, and prediction model or predict. Due to the absence of a consensus definition of AKI before 2010,^15^ four English and four Chinese databases were systematically searched from January 1, 2010, to June 6, 2022, including Cochrane Library, Embase, Medline (PubMed), Web of Science, Wan Fang, VIP, CNKI, and Sinomed, with no limitation in publication type. The detailed search strategy is shown in Table S1. The literature was screened by two investigators based on titles and abstracts, and full articles were reviewed if eligible, with disagreements resolved by a third reviewer. All studies pertaining to the development, updating, or validation of prognostic multivariable models for AKI in hospitalized children were included, either as cohort studies or case‐control studies. The detailed inclusion and exclusion criteria are shown in Table S2.

### Data extraction and study quality assessment

A data extraction form was developed based on the CHARMS checklist^23^ and previous reviews^18^ (Table S3), which included detailed information on the design, population, location, outcome, modeling method, method of internal validation, number of participants and events, number and type of predictors, model presentation and predictive performance in individual studies.^18^ Considering the particularity of children, the information about the handle of SCr and age‐dependent variables was paid more attention to. The presence of external validation and model impact studies were recorded.

Quality assessments of all studies were performed by two investigators in two aspects: 1) transparency of the reporting; and 2) ROB. According to the TRIPOD statement (37 items in total), prognostic prediction models were classified into six types: 1a, 1b, 2a, 2b, 3, and 4 (Figure S1 and Table 1), and the transparency of the reporting in the literature was evaluated by a global TRIPOD score, i.e., the sum of the scores for each individual item (out of a maximum of 37, with a score of 1 if the was criterion met, and a score of 0 for each item that did not meet the criteria, or if it was unclear).^18^, ^25^ Unlike other quality assessment tools, such as QUIPS (quality in prognosis studies), QUADAS‐2 (quality assessment of diagnostic accuracy studies), ROBINS‐I (ROB in nonrandomized studies of interventions), and Cochrane Bias risk Assessment Tool 2.0, the PROBAST tool was specially designed for use in systematic reviews of prediction model studies and it also was used as a general tool for critical appraisal of (primary) prediction model studies, which assesses ROB and applicability of primary prediction model studies.^28^ The ROB and the applicability of the prediction model in this study were assessed based on the PROBAST guideline which assessed the ROB in four domains (participants, predictors, outcome, and analysis) and applicability to the intended population and setting in three domains (participants, predictors, outcome).

**TABLE 1:** Basic characteristics of the included studies

Population	Author, year	Country	TRIPOD type	N‐all	N‐event	Event with a time span of prediction	AKI diagnostic criteria	Baseline SCr^†^
Critically ill children in ICU	Dong, 2021^40^	USA, UK	2a	16 863	1922	Severe AKI during the timeframe 48 to 6 h before AKI onset	KDIGO	a. Mean normal
	Wang, 2017‐1^35^	USA	3	2198	1216	any AKI during 1 day prior to admission to discharge	KDIGO	b. 90–7d Lowest
	Basu, 2014^33^	USA	3, 4	584	80	Severe AKI within 72h of ICU stay	KDIGO	b & c. Calculated by Schwartz methods, or 90d lowest
	Sanchez‐Pinto, 2016^34^	USA	3	9396	377	any AKI within 72h of ICU stay	KDIGO	a & b. 90d most recent or low normal
	Sanchez‐Pinto, 2018^36^	USA	2a					
Non‐ICU	Wang, 2017‐2^35^	USA	3	3811	1191	any AKI during 1 day prior to admission to discharge	KDIGO	b. 90–7d lowest
CLD	Vijay, 2020^39^	Indian	1a	247	41	any AKI	KDIGO	/
Septic shock	Stanski, 2020^38^	USA	3	840	167	Severe SA‐AKI within 72h of ICU stay	KDIGO	c. Calculated by Schwartz methods or Pottel method
VLBW Infants	Hu, 2020^37^	China	1b	604	144	any AKI	KDIGO	b. 7d lowest after birth
Total	‐	‐	‐	34 543	5138	‐	‐	‐

Abbreviations: AKI, acute kidney injury; CLD, chronic liver diseases; ICU, intensive care unit; N‐all, the sample size in derivation and validations; N‐event, the number of outcomes in all cohorts; SA‐AKI, severe sepsis‐associated AKI; SCr, serum creatinine; Severe AKI, stage 2 and above AKI; TRIPOD, Transparent Reporting of a multivariable prediction model for Individual Prognosis or Diagnosis; VLBW Infants, very low birth weight infants.

^†^ The definition of the baseline SCr included three types: a. the normal value for age and gender group; b. the historic value before admission; c. the value calculated by the formula (Mean normal = the mean normal SCr for age and gender group; Low normal = the low normal SCr for age and gender group; 90d Lowest = the lowest in the 90 days before admission; 90–7d lowest = the lowest SCr obtained 90 days before through the first week of admission; 90d most recent = the most recent SCr obtained in the 90 days before admission; 7d Lowest after birth = the lowest level recorded previously since the baseline level changes constantly during the first week of birth; Calculated by Schwartz methods = imputed based on the estimated creatinine clearance and the patient height using the Schwartz correction; Calculated by Pottel methods = base on the age‐based Pottel method).

### Outcome, model performance, and clinical utility

Outcomes were the occurrence of AKI defined by diagnostic criteria such as the p‐RIFLE,^12^ AKIN,^12^ KDIGO,^14^ or p‐ROCK^6^ criteria, regardless of the stage of AKI (Table 1). This review collected information on the definitions of baseline renal function, whether SCr was used as a predictor in the analysis, as well as the time span used to define the outcome (Table 1). If there are significant differences in the diagnostic criteria for AKI, the subgroup analysis should be performed according to the outcome definition. The model performance was evaluated from two perspectives: discrimination and calibration.^29^ The discrimination represented the ability of a model to separate and rank patients with an outcome from those without an outcome, usually assessed by the area under the receiver operating characteristic curve (AUC) or C‐Statistic.^30^, ^31^ The AUC more than 0.8 was considered to have good discrimination, while the AUC more than 0.9 was considered to have very good discrimination.^30^ The calibration was assessed by the Hosmer‐Lemeshow (H‐L) test or calibration plot of expected and actual outcomes,^32^ which describes how the well‐predicted results agree with the observed results and goodness of fit.^30^ The clinical utility was recorded, for example, ease of bedside use and whether the models could be electronically automated.^20^ In addition, model impact studies, which evaluated the models on clinical practice, were investigated by searching the cited articles of the included literature in the Web of Science database and Research Gate platform. Considering the long publication cycle of relevant impact articles, we contacted authors via email to ask whether the existing model has been evaluated based on randomized controlled trials and whether the model has been used during the actual clinical decision‐making process.

## RESULTS

### Characteristics of included studies

**FIGURE 1:**
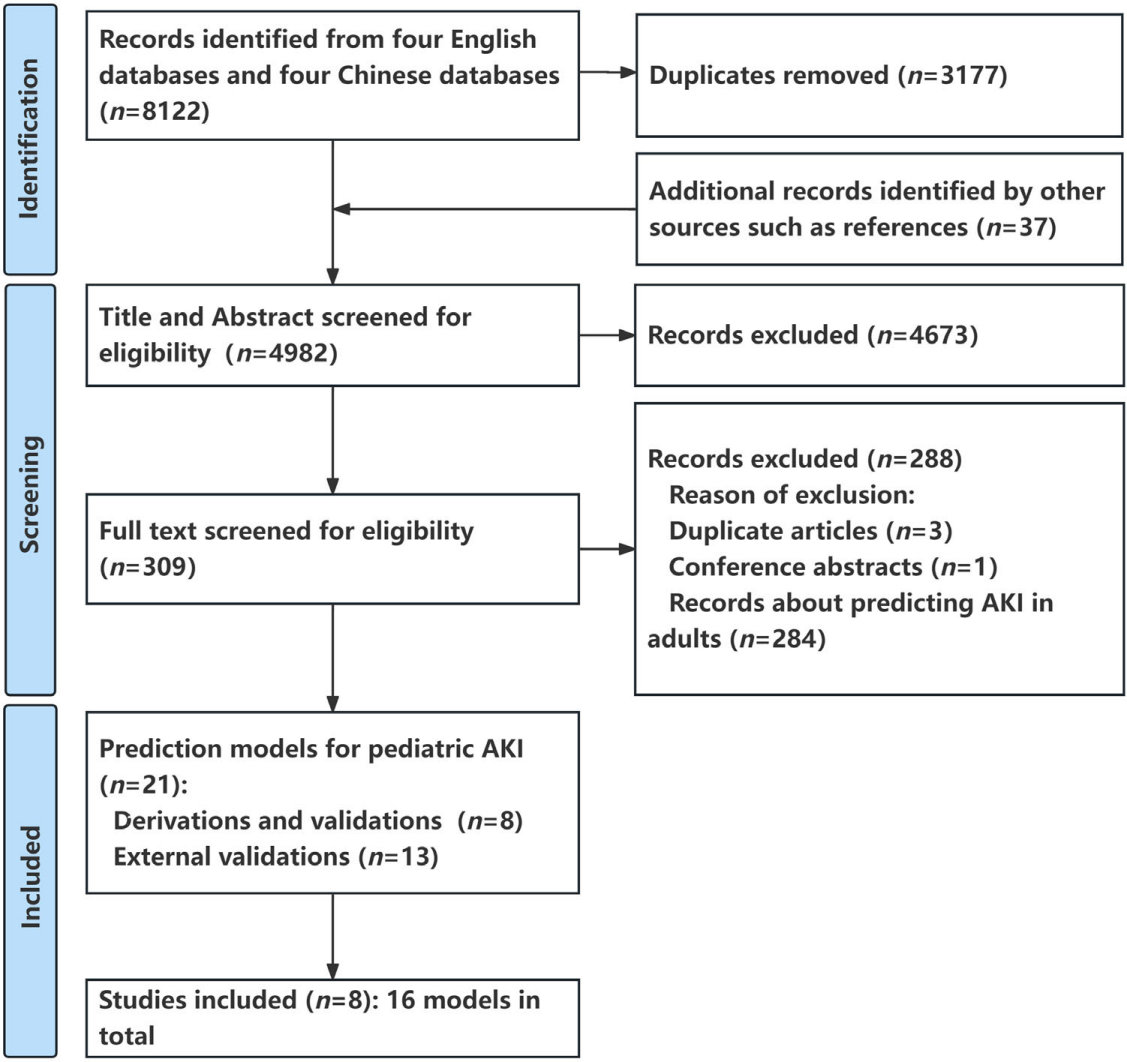
The Preferred Reporting Items for Systematic Review and Meta‐Analysis (PRISMA) study flow chart; AKI, acute kidney injury.

The PRISMA study flow chart is shown in Figure 1. Overall, 8122 potentially eligible records were identified by eight databases. A total of 3177 duplications, marked as ineligible by automation tools of EndNote software, were excluded, and 37 additional records identified from references were added to format the initial research pool (*n* = 4982). After screening titles and abstracts, 4673 records were excluded according to the inclusion and exclusion criteria. Then, the full‐text evaluation was carried out for the remaining 309 records. Among them, 21 studies reported the prediction models for AKI in hospitalized children (eight articles regarding the development and validation of original models,^33^, ^34^, ^35^, ^36^, ^37^, ^38^, ^39^, ^40^ and 13 articles regarding the external validation studies^41^, ^42^, ^43^, ^44^, ^45^, ^46^, ^47^, ^48^, ^49^, ^50^, ^51^, ^52^, ^53^), while another 288 studies were excluded (three duplicate articles, one case report/meeting abstract, as well as 284 articles regarding predictions of AKI in adults). Finally, eight articles that, reported the original model development were included in order to extract data, in which there were 16 models (12 models for critically ill children who were admitted to the intensive care unit [ICU],^33^, ^34^, ^35^, ^36^, ^40^ one model for general pediatric patients in non‐ICU,^35^ one model for children with chronic liver diseases [CLD],^39^ 1 models for children with septic shock,^38^ and one model for very low birth weight [VLBW] infants^37^). Six studies were retrospective cohorts^33^, ^34^, ^35^, ^36^, ^37^, ^40^ and two were prospective observational studies.^38^, ^39^ The United States (*n* = 6) accounted for the majority of the models,^33^, ^34^, ^35^, ^36^, ^38^, ^40^ with five studies coming from a single center.^34^, ^35^, ^36^, ^37^, ^39^ The AKI incidence in hospitalized children was 14.9% (5138 events), which varied according to different diseases or different clinical departments, in which heterogeneous definitions were employed (Table 1 and Table S4). The patients without AKI episodes were older than those with AKI episodes in two studies.^35^, ^38^ Mortality was reported to be significantly higher in those who developed AKI in two studies,^34^, ^38^ ranging from 26% to 37% (Table 2).

**TABLE 2:** Basic characteristics, model performance, and clinical applicability of acute kidney injury (AKI) prediction models in children

Variable	Critically ill patients in ICU							
	Dong, 2021^40^ (*n* = 14 334)	Wang, 2017‐1^35^ (*n* = 1332)	Basu, 2014^33^ (*n* = 144)	Sanchez‐Pinto, 2016^34^ (*n* = 3932)	Sanchez‐Pinto, 2018^36^ (*n* = 3932)			
					Model‐1	Model‐2	Model‐3	Model‐4
Centers, design	3, R	1, R	19, R	1, R	1, R	1, R	1, R	1, R
Modeling method	EML	LR	RAI	LR	LR (*P*‐value)	LR (AIC)	LR (Lasso)	LR (EN)
Age (with outcome, years)	−	2.6 (0.6, 10.5)^b^	−	−	−	−	−	−
Age (no outcome, years)	−	6.5 (1.4, 13.6)^b^	−	−	−	−	−	−
Events (derivation)	653	791	28	161	161	161	161	161
Predictors tested	35	29	5	34	26	26	26	26
Predictors included	16	10	5	7	11	13	23	23
EPP	18	27	5	4	6	6	6	6
Sample size of EV	−	866	118, 108, 214	2632, 2832	−	−	−	−
Derivation AUC	0.89	0.75	0.77	0.84	0.837	0.826	0.832	0.833
IV AUC	0.88, 0.78, 0.90	0.74	−	0.81	−	−	−	−
EV AUC	−	0.74	0.74, 0.80, 0.81	0.86	−	−	−	−
Derivation calibration	−	Plot	−	H‐L	−	−	−	−
IV calibration	Plot	Plot	−	H‐L	−	−	−	−
EV calibration	−	Plot	−	H‐L	−	−	−	−
TRIPOD items reported	22	28	27	30	15	15	15	15
Bedside calculation	Yes	Yes	−	−	−	−	−	−
Electronic automation	Yes	Yes	−	−	−	−	−	−
Model impact studies^a^	−	Yes	−	−	−	−	−	−

Variable	Critically ill children in ICU				Non‐ICU	CLD	Septic Shock	VLBW Infants
	Sanchez‐Pinto, 2018^36^ (*n* = 3932)				Wang, 2017‐2^35^ (*n* = 2337)	Vijay, 2020^39^ (*n* = 247)	Stanski, 2020^38^ (*n* = 379)	Hu, 2020^37^ (*n* = 604)
	Model‐5	Model‐6	Model‐7	Model‐8				
Centers, design	1, R	1, R	1, R	1, R	1, R	1, P	14, P	1, R
Modeling method	RF (VSURF)	RF (Boruta)	RF (RRF)	RF (GBFS)	LR	LR	CARTM	LR
Age (with outcome, years)	−	−	−	−	5.3 (2.0, 12.4)^b^	8 (2.6, 13.4)^b^	5.1 (1.3, 13.5)^b^	2.4 (0.5, 70.3)^c^
Age (no outcome, years)	−	−	−	−	10.6 (3.3, 15.3)^b^	−	6.5 (2.5, 12.4)^b^	2.3 (0.3, 72)^c^
Events (derivation)	161	161	161	161	722	41	65	144
Predictors tested	26	26	26	26	29	3	5	22
Predictors included	9	17	20	9	8	3	5	6
EPP	6	6	6	6	24	13	13	6
Sample size of EV	−	−	−	−	1474	−	691	−
Derivation AUC	0.809	0.824	0.817	0.785	0.69	0.926	0.95	0.79
IV AUC	−	−	−	−	0.69	−	0.88	0.788
EV AUC	−	−	−	−	0.69	−	0.83	−
Derivation calibration	−	−	−	−	Plot	−	−	H‐L (*P* = 0.245)
IV calibration	−	−	−	−	Plot	−	−	−
EV calibration	−	−	−	−	Plot	−	−	−
TRIPOD items reported	15	15	15	15	28	16	28	27
Bedside calculation	−	−	−	−	Yes	−	−	−
Electronic automation	−	−	−	−	Yes	−	−	−
Model impact studies^†^	−	−	−	−	Yes	−	−	−

Abbreviation: AIC, Akaike Information Criterion; AKI, acute kidney injury; AUC, area under the receiver operating characteristic curve; CARTM, classification and regression tree methodology; CLD, chronic liver diseases; EML, ensemble machine learning; EN, elastic net; EPP, events per predictor; EV, external validation; GBFS, gradient boosted feature selection; H‐L, Hosmer‐Lemeshow test; IV, internal validation; Lasso, least absolute shrinkage and selection operator; LR, logistic regression; P, prospective observational study; R, retrospective cohort study; RAI, renal angina index; RF, random forest; RRF, regularized random forests; TRIPOD, Transparent Reporting of a multivariable prediction model for Individual Prognosis or Diagnosis; VLBW, very low birth weight; VSUFR, variable selection using random forest; “−” referred to that information cannot be obtained from the articles.

^a^ Model impact studies refer to the reporting that evaluates the model on clinical practice. The “Yes” means that the model impact study of the prediction model for pediatric AKI has been reported.

^b^ Median (interquartile range);

^c^ Median (range), the age is shown in hours.

### Quality assessment and ROB

**FIGURE 2:**
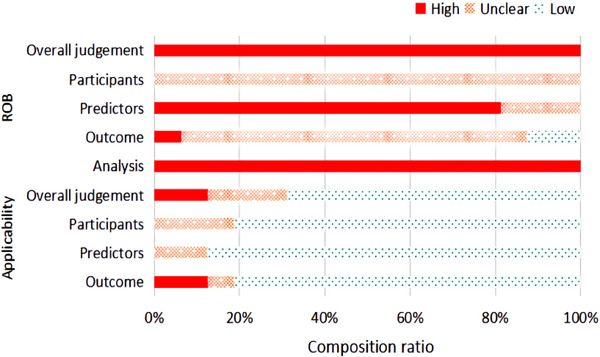
The constituent ratio of studies with different levels of ROB in each domain. ROB, risk of the bias; Red = ‘high’ risk of bias, green = ‘low’ risk of bias, or amber = ‘unclear’ risk of bias.

A median of 27 (interquartile range [IQR] 21–28) of 37 recommended items were reported, manifesting significant limitations (key limitations in Table S5, TRIPOD reporting in Table S6), including the means of dealing with missing value, the calculation of sample size, the blinding method used in data collection, selection of methods of predictors, the use of certain validation techniques, and the updating of models. For example, only three studies reported imputation techniques for missing data.^33^, ^34^, ^36^ None of the studies reported sample calculation or blinding methods for their respective collecting predictors or outcomes. Table 2 lists the level of ROB of each study in different domains, and Figure 2 shows the constituent ratio of studies with different levels of ROB in each domain. Based on the PROBAST tool, the results suggested that all included studies were at a high ROB (Figure 2, Table 3, and Table S7) with shortcomings across the major domains of the assessment (Table S5). More details of the assessment of PROBAST are as follows: As for predictors' domains, all studies did not mention the blinding method in data collection, so information bias in research is unavoidable. In the analysis domain, 11 of 16 models had less than 10 events per predictor (EPP) assessed which caused potentially insufficient power and increased the risk of model overfitting.^33^, ^34^, ^36^, ^37^

### Candidate predictors, sample size, and model building

A median of 26 (IQR 25–27) predictors were mentioned, though most studies only reported those included in the final models. A variety of predictors for model training were considered using univariate analysis in 13/16 of the models.^34^, ^35^, ^36^, ^37^, ^40^ Of the 60 different predictors, a median of 10 (7–16) per model was included, including demographics, diagnosis, medicines, laboratory indexes, as well as others (Figure S2 and Tables S8–S10). The ways of handling the variables with age‐dependent trends were not reported clearly and coincidently. Three studies used age‐normalized to address predictors with age‐dependent trends,^34^, ^35^, ^36^ that is, the quantitative variables were classified as low, normal, and high according to the RIs for children by age group. Only one study used an age‐dependent ensemble machine learning model.^40^ Two studies included SCr rate of change as a predictor in the final model^33^, ^40^ and one study included AKI status at admission.^38^ The handling of SCr was included when a baseline renal function was calculated (prior or at admission); when SCr was used as a predictor is summarized in Table 1 and Table S4. Nine models mentioned the shrinkage techniques of variables,^36^, ^40^ but none of the models calculated the sample size. Median number of outcome events was 161 (65–722). For statistical power, 12 models had more than 10 EPP included in the final model (16 models in total). However, the EPP was < 10 in 11 models,^33^, ^34^, ^36^, ^37^ when accounting for the total number of candidate predictors assessed. In hospitalized children, new modeling techniques such as recurrent neural network model^54^ were not described. Six of the eight included studies used logistic regression during modeling.^34^, ^35^, ^36^, ^37^, ^38^, ^39^

### Outcome, model performance, and clinical utility

The diagnostic criteria of outcomes in all included studies were uniformly based on the KDIGO criterion. As for the time span of prediction, which means the time period from admission to the occurrence of outcomes, was during the 72‐h period after admission in four studies,^33^, ^34^, ^36^, ^38^ one day prior to admission to discharge in one study,^35^ however not described clearly in two studies.^37^, ^39^ Only one study predicted the AKI during the time span of 48–6 h before AKI onset.^40^ The definitions of the baseline SCr included three types: the normal value for age and gender group; the historic value before admission; and the value calculated by the formula. With that said, only one study considered within‐hospitalization values as the baseline (Table 1 and Table S4).^35^ There was no information regarding prior episodes of AKI in the all included studies.

As for model performance, the median AUC in derivation was 0.83 (range 0.69–0.95) among 16 models in which only two models were considered to have very good discrimination.^38^, ^39^ Only two studies presented a calibration plot for derivation and validation.^35^, ^40^ The H‐L test was used in two derivations and one internal validation.^34^, ^37^ Five models have been externally validated, with regards to separate populations within the same study or other model studies, where the sample size ranged from 108 to 2832 and the median AUC was 0.79 (range 0.69–0.86), in which two model were considered to have a good discrimination.^34^, ^38^ Two external validations provided a calibration plot,^35^ and one reported the H‐L test.^35^ In the Wang external validation, the calibration suggested the model for ICU and non‐ICU children, which indicated a good model fit.^35^

Regrettably, only three models allowed easy bedside calculation or potential electronic automation (the AKI risk calculator in Wang's study, and intelligently engineered predictors automatically assessing real‐time AKI risk in Dong's study)^35^, ^40^ Only two models reported the effectiveness of clinical application by the randomized controlled trials,^35^, ^55^ which remaindered that the alert based on real‐time risk prediction of AKI increased screening rates of SCr testing in ICU, but did not decrease the AKI incidence or severity in pediatric ward settings.^55^ In addition, we contacted the investigators but did not receive a response.

## DISCUSSION

### Principal findings

Currently, researchers are devoted to developing prediction models to early detect pediatric AKI, however, their levels of quality are limited and the models rarely are applicable in clinical practice. The results of this review showed that the generalization ability of most models was limited by either the lack of external validation, the model impact studies or evidence of clinical implementation, incomplete reporting, and little consideration of electronic automation. Besides the modeling algorithm, this review found that the challenges for developing prognostic prediction models for pediatric AKI are reflected by the reporting deficiencies included in ways of determining baseline SCr. Also, it is concerned with age‐dependent blood biochemical indexes for pediatric participants. In addition, few prognostic prediction models for pediatric AKI were performed for external validation, let alone the transformation in clinical practice.

PROBAST suggests that all studies have domains that place the studies at high ROB; the main reasons are insufficient reporting and methodological shortcomings. For example, no study collected predictors or outcomes in blinding methods, thus information bias was unavoidable. Although the phenomenon of under‐reporting improved after the formation of the TRIPOD statement,^25^ there were still insufficient reports regarding dealing with missing values, calculating the sample size, the blinding method in data collection, as well as selecting the method of predictors (Table 3), which resulted in an unclear ROB. Meanwhile, it was difficult to assess the impact of high ROBs on model prediction effect in our study due to the lack of information on the application of these models in clinical scenarios. Due to all studies having high ROB in our study, we thought all included models for predicting AKI in children were not the best model and prognostic prediction models for pediatric AKI need further establishment. The above analysis remains that the factors that led to high ROB should be avoided in the research design stage and the reporting of predictive model research should be strictly in accordance with standardized reporting standards such as the TRIPOD statement.

**TABLE 3:** Risk of bias summary based on Prediction study Risk of Bias Assessment Tool (PROBAST)

Reference	ROB				Applicability			Overall judgement	
	Participants	Predictors	Outcome	Analysis	Participants	Predictors	Outcome	ROB	Applicability
Dong, 2021^40^	？	‐	‐	‐	+	+	‐	‐	‐
Wang, 2017‐1^35^	？	‐	？	‐	+	+	+	‐	+
Basu, 2014^33^	？	？	‐	‐	+	+	‐	‐	‐
Sanchez‐Pinto, 2016^34^	？	？	+	‐	+	+	+	‐	+
Sanchez‐Pinto, 2018^36^	？	‐	？	‐	+	+	+	‐	+
Wang, 2017‐2^35^	？	‐	？	‐	+	+	+	‐	+
Vijay, 2020^39^	？	‐	？	‐	？	+	+	‐	？
Stanski, 2020^38^	？	‐	？	‐	？	？	？	‐	？
Hu, 2020^37^	？	？	？	‐	？	？	+	‐	？

‘‐’ = ‘high’ risk of bias, ‘+’ = ‘low’ risk of bias or ‘？’ = ‘unclear’ risk of bias.

Abbreviations: CLD, chronic liver diseases; ROB, risk of bias; VLBW Infants, very low birth weight infants.

As for the methodological shortcomings shown in Table S5, a predominantly insufficient sample size had the potential to lead to overfitting. A few studies mentioned that shrinkage techniques make prediction models easy to operate, and utilizing imputation to handle missing data increases sample size and power. Handling of SCr was of particular concern in a number of areas. However, the included studies had heterogeneous or unclear definitions of the magnitude of SCr rise and time span. A part of the studies included SCr rate of change^33^, ^40^ or AKI status at admission^38^ as predictors in the final model, thus potentially confusing prediction with a diagnosis of AKI.^24^ Furthermore, several studies conducted external validation, particularly a few discrimination and calibration tests, in addition to further research techniques such as decision curve analysis which can offer insight into clinical consequences.^56^

Based on the aforementioned analysis, the challenges for developing models for pediatric AKI are summarized as follows:

Firstly, it is complex but significant to determine the befitting outcome for the study population. For example, any stage AKI is commonly used in general hospitalized patients,^35^ but it is more appropriate to select cases of severe AKI or RRT for critically ill patients in ICU.^40^ The KDIGO diagnostic criterion for AKI requires a clear baseline SCr as a reference. One difficulty is how to define the baseline SCr properly due to the high variability of SCr in children.^21^, ^22^ Some researchers believed that the modified KDIGO definition based on the Schwartz method may be the preferable approach for diagnosing AKI in critically ill children,^57^ but no universal consensus exists regarding which definition to use in pediatrics.^57^ Meanwhile, the prognostic prediction model should be able to predict the occurrence of AKI in advance to give doctors enough time for treatment.^15^ Therefore, not only the diagnostic criterion but also the time span to define AKI, plays a significant role in modeling. According to the 15th International Consensus Conference of the Acute Dialysis Quality Initiative (ADQI) Group, the time span is at least 72 h,^15^ and it may be a good choice to establish a continuous prediction of future AKI with new modeling techniques based on the electronic health records.^15^, ^54^, ^58^

Secondly, the challenges are reflected by the limitations of modeling algorithms. A majority of studies selected variables for multivariate analysis based only on univariate analysis, but the ADQI Group recommended that risk factors that are well established in the literature should be considered along with novel risk factors identified via machine learning techniques.^15^ Besides the methodological shortcoming mentioned above, the trickier issue is the inherent shortcoming of the modeling algorithm. The value of machine learning for AKI prediction lies in the ability to handle large amounts of data and to identify seemingly benign relationships with those data as they relate to disease onset. However, machine learning methods usually have black box problems of predictors.^40^ For instance, new modeling techniques such as recurrent neural networks or long short‐term memory networks models are more well‐suited to make predictions with time series data than logistic regression for continuous prediction of future AKI,^15^, ^54^, ^59^, ^60^ whereas the importance of predictors should be further explored using SHAP package to improve clinical interpretability.

Thirdly, external validations focus on the transportability and generalizability of the model, which can improve the quality of research results and make the prediction model more credible. However, it is a typical challenge to find appropriate datasets with a large enough sample size and appropriate subjects for external validation studies in hospitalized children. Generally, external validation may use four different participant datasets: 1) collected by the same investigators from a later period; 2) collected by other investigators in another hospital or country; 3) in similar participants from an intentionally different setting, or 4) in other types of participants.^25^ Our study found that performance validation of participant data, collected from a later period, may be better than the others, but only represents the temporal validation which is considered intermediate between internal and external validation.^25^ In case of poor performance, when evaluated using the last three types of external validation datasets, the model can be updated or adjusted.^25^

Fourthly, how to promote prediction model use in clinical practice is an equally important challenge. Ideally, AKI risk prediction tools should directly extract relevant data predictors in real‐time, deliver a relevant “renal risk score” and provide feedback to practitioners regarding potentially actionable items.^15^ However, only three models in our study allowed easy bedside calculation or potential electronic automation. Thus, it is difficult to transform these models into clinical practices and surveillance of disease. The expert consensus at the 15th ADQI conference proposed that the models should be simplified for bedside use or that any prototypes should be directly integrated into the electronic health record for automated real‐time usage instead of staying in the stage of simple prediction models.

Finally, the challenges of developing pediatric‐specific prognostic prediction models the challenges are reflected by the handling of age‐dependent blood biochemical indexes for pediatric participants. The handling of the variables, which vary significantly with age was not clear and coincident. The commonly used method of handling the variables was the age‐normalized method, that is, continuous variables were classified into categorical variables (normal, too high, and too low) according to the RIs of blood biochemical indexes grouped by age, which could consequently result in the loss of original information. A recent study established an age‐dependent ensemble machine learning model for pediatric AKI and made a sophisticated lookup table based on the patient's age and single predictor value, which could be the more appropriate method than the traditional age‐normalized method to decrease the impact of age on other predictors in pediatric patients.^40^

### Strengths and limitations of this review

Regarding its strengths, it is the first systematic review to critically appraise prognostic prediction models for AKI in hospitalized children (PRISMA Checklist is shown in Table S11). The current research status and clinical application of AKI risk prediction models in the pediatric field are well summarized from several perspectives by searching the model impact studies and by contacting the respective authors. However, there are some limitations in this research: 1) Due to a lack of meta‐analyses of different prognostic models,^27^ this study was forced to be conducted as a qualitative descriptive analysis; 2) Considering that there were no unified diagnostic criteria for AKI before 2010,^15^ the search period was limited to between 2010 and 2022. Meanwhile, the multi‐database search strategy was limited to titles, keywords, and abstracts, which may lead to missed detection; 3) The information collected is based largely on peer‐reviewed published scientific literature, which is subject to unpublished bias; 4) There is a non‐response bias in the clinical application evaluation survey of existing models.

### Comparison with previous systematic reviews

This review and the previous reviews include adherence to recommended TRIPOD reporting with similar disadvantages.^18^ Considering both the frequencies of predictors in prediction models and studies, the most common predictors included age, platelet count, blood urea nitrogen, pH, and total bilirubin in this review. Compared with a systematic review in adults, this article found that predictors of high frequency were very different between children and adults,^18^ possibly because of the differences between adults and children in terms of disease spectrum and drug use spectrum. For example, cardiovascular drugs are commonly used in adults,^18^ while children are mainly exposed to ampicillin, acyclovir, and other antibacterial and antiviral drugs.^61^ Diabetes mellitus and heart failure are common diseases in adults, while rare in children.^18^ This indicates that the adult prediction model is not suitable for children, and it is significant to develop AKI prediction models in the pediatric population. Meanwhile, some well‐known factors that increase the risk of AKI, such as nephron endowment, renal development issues during pregnancy, and antibiotics exposure,^62^, ^63^, ^64^ are not reported in all previously published studies, which may be due to the fact that these factors are not widely available in clinical examination.

### Conclusions and future directions

Besides the modeling algorithm, the challenges for developing prediction models for pediatric AKI reflected by the reporting deficiencies included ways of handling baseline SCr and age‐dependent blood biochemical indexes. Moreover, few prediction models for pediatric AKI were performed for external validation, let alone the transformation in clinical practice. Further investigation should focus on the combination of prediction models and electronic automatic alerts. The generalization ability of most models was limited by either the lack of external validation, the model impact studies or evidence of clinical implementation, incomplete reporting, and little consideration of electronic automation. Therefore, considering the large differences in the clinical treatment guidelines set by different hospitals, the models should be updated in order to be widely used. For example, it was a potential strategy to improve the clinical applicability of models to simplify the model for bedside use or integrate them into electronic health record systems. The research regarding combining the prediction model with the early warning device needs to be further investigated.^15^ Moreover, the evidence of good model performance in external validation is interesting, however, it does not necessarily equal the good clinical application value.^65^ In the future, the value of clinical application needs to be further evaluated through the impact study, which focuses on improving the incidence of AKI or other prognostic indicators.

## CONFLICT OF INTEREST

The authors declare no conflict of interest.

## Supporting information



Supporting Information
