# Supplementary material for: The challenges for developing prognostic prediction models for acute kidney injury in hospitalized children: A systematic review
Source: Pediatr Investig. 2024 Dec 11;9(1):70–81. doi: 10.1002/ped4.12458 (PMC11998178; doi:10.1002/ped4.12458)
Supplement: Supplementary file 1 — Supporting Information [file PED4-9-70-s001.pdf]

Supplementary Material for

The challenges for developing prognostic prediction models for acute kidney injury in hospitalized children: A systematic review

Chen Wang, Xiaohang Liu, Chao Zhang, Ruohua Yan, Yuchuan Li, Xiaoxia Peng

Content

Table S1. Search strategy 2

Table S2. Study inclusion and exclusion criteria 3

Table S3. CHARMS checklist and data extracted for systematic review 4

Table S4. Full details of models reviewed 5

Table S5. Limitations in methodology and reporting 10

Table S6. TRIPOD items reported in the 8 studies 11

Table S7. Risk of bias evaluation rules based on Prediction study Risk Of Bias Assessment Tool .. 12

Table S8. Most common predictors used in the 16 models 13

Table S9. All predictors included in the 16 models 14

Table S10. All predictors included in the 8 studies 16

Table S11. PRISMA 2020 Checklist 18

Fig. S1 Types of prediction model studies in TRIPOD statement 20

Fig. S2 The most common predictors in prediction models for AKI in hospitalized children 21

Table S1. Search strategy

Embase

('acute kidney injur*':ti OR 'acute renal injur*':ti OR 'acute renal injuries':ti OR 'acute kidney failure*':ti OR 'acute renal failure*':ti OR 'acute kidney insufficienc*':ti OR 'acute renal insufficienc*':ti OR 'acute kidney dysfuction*':ti OR 'acute renal dysfuction*':ti OR 'acute kidney disease*':ti OR 'acute renal disease*':ti) AND predict*:ti AND [2010-2023]/py **2299**

Cochrane Library

#1: MeSH descriptor: [Acute Kidney Injury] explode all trees

#2: (predict*):ti,ab,kw

#3: #1 and #2 with Cochrane Library publication date from Jan 2010 to Jun 2022 **121**

Web of Science

(TI="acute kidney injury" OR TI="acute renal injury" OR TI="acute kidney injuries" OR TI="acute renal injuries" OR TI="acute kidney failure*" OR TI="acute renal failure*" OR TI="acute kidney insufficiency" OR TI="acute renal insufficiency" OR TI="acute kidney insufficiencies" OR TI="acute renal insufficiencies" OR TI="acute kidney dysfuction*" OR TI="acute renal dysfuction*" OR TI="acute kidney disease*" OR TI="acute renal disease*") AND TI=predict* 2010-01-01 to 2022-06-06 (Publication date) **2149**

Medline (PubMed)

(acute kidney injury[MeSH Terms]) AND (predict*[Title]) AND (2010:2022[pdat]) **1588**

Sinomed (Chinese database)

#1: "急性肾损伤"[不加权:扩展]

#2: "预测"[标题:智能] or "预警"[标题:智能] or "风险"[标题:智能]

#3: #1 or #2 年代 2010-2022 **411**

CNKI (Chinese database)

(TI=急性肾损伤 OR TI=急性肾衰竭 OR TI=AKI OR TI=ARF) And (TI=预测 OR TI=预警 OR TI=风险) And (发表时间 Between('2010-01-01' and '2022-06-06') **539**

Wang fang (Chinese database)

(题名:(急性肾损伤) or 题名:(急性肾衰竭) or 题名:(AKI) or 题名:(ARF)) and (题名:(预测) or 题名:(预警) or 题名:(风险)) And Date:2010-2022 **538**

VIP (Chinese database)

（T=急性肾损伤 OR T=急性肾衰竭 OR T=AKI OR T=ARF） and （T=预测 OR T=预警 OR T=风险） And 年份：2010-2022 **432**

Table S2. Study inclusion and exclusion criteria

Inclusion Criteria

- Articles in peer-reviewed journals reporting a multivariable prediction model (scoring system or algorithm) identifying pediatric patients (age ＜21year) who developed AKI
- Retrospective or prospective cohorts and case control studies
- Updating and validation studies of an existing model
- Articles published after 2010

- **Exclusion Criteria**
- Articles about non-population trials, such as animal experiments
- Only logistic regression analysis without a prediction model
- Only validation studies of an existing model
- Studies that investigated a single predictor, test, or biomarker which are novel and not available in clinical examination
- Studies that only investigated causality between one or more predictors and an outcome
- Studies included the population already with the outcome (e.g., Severe AKI present at the time of prediction).
- Case reports, conference abstracts, reviews or comments were excluded

Abbreviation: Area under the receiver-operating characteristic curve, AUC; Acute kidney injury, AKI.

Table S3. CHARMS checklist and data extracted for systematic review

Item	Explanation in the Review
**1. Type of studies**	Prognostic prediction models
**2. Scope**	Published prognostic prediction models for development of AKI in general hospital settings; to inform risk stratification and potential uses in decision-making in different patient groups
**3. Type of studies**	Model development with or without external validation in independent data; external model validation and model updating, if present
**4. Target population**	Pediatric Patients (age＜21 years old) in hospital settings
**5. Outcome to be predicted**	Development of AKI (defined by p-RIFLE, AKIN, pROCK, or KDIGO criteria) after an admission to hospital
**6. Time span of prediction**	In-hospital development of the outcome
**7. Intended moment of using the model**	At admission to risk stratify or guide therapy

Summary of Data extracted
• Data source (years; retrospective, prospective; cohort, case-control)
• Participants and setting (e.g., Department in the hospital, single or multi-center, country, if with special disease)
• Outcome (Definition, method for measurement with any blinding, time of outcome occurrence)
• Candidate predictors (Number, definitions, handling of predictors in the modelling)
• Sample size, EPV (including all predictors considered)
• Type of model evaluation - derivation, validation (internal or external)
• Missing data (number, handling of the missing data)
• The handling of SCr and variables which vary significantly with age
• Model development (e.g., modelling methods, method for selection of predictors, shrinkage technology)
• Incidence of outcome and mortality data
• Predictors included in the final models
• Model performance: discrimination (AUC or C-Statistic), calibration (e.g., H-L P value, slope/curve)
• Internal and external validation (in same study)
• External validation studies with relevant performance measures

Abbreviation: Acute kidney injury, AKI; Events per variable, EPV; Hosmer-Lemeshow goodness-of-fit test, H-L.

Table S4. Full details of models reviewed

Critically ill patients in ICU

Author, Type, TRIPOD detail	Population, Outcome, AKI Definitions, Baseline Scr, Methods	Outcomes, Predictors & Model performance	External Validation
**Dong et al, 2021**<br><br>**PICU and CTICU**<br><br>**TRIPOD 2A – Derivation, IV (22/37 pts)**	USA and UK multi-center (3) retrospective cohort study. Data were collected from EHRs in 3 hospitals. 2003-2011 (Hospital1, USA), 2009-2015 (Hospital2, UK), 2014-2019 (Hospital3, USA). Mean Age 4 [0.7, 12.1], 2 [0.6, 6.0] and 7 [3.8, 14.7], female 46%, 46% and 48% in 3 hospitals.<br><br>Inclusion: patients from the PICU and CTICU of three independent tertiary-care pediatric intensive care center.<br><br>Exclusions: (1) patients below 1month (neonatal), above 21 years, or without a valid age record, (2) patients with AKI in the first 12 h of ICU stay, (3) patients with length of stay less than 24 h, (4) if a single patient had multiple encounters, only one encounter was used while remaining encounters were excluded. Included: n=16,863<br>Outcome: Stage 2/3 AKI during the timeframe 48 to 6 h before onset (KDIGO SCr criteria). Any AKI (Stages 1/2/3) and prediction of requirement of RRT using KDIGO SCr criteria. The Baseline SCr was the mean normal creatinine level for age and gender group.<br>Candidate predictors were selected in a two-step process. In the first step, predictors were filtered to remove co-linearity while keeping those most strongly associated with the AKI label. Among multiple predictors determined to be co-linear (correlation higher than 0.97), only the predictor having highest mutual information with the AKI label was kept (250 candidate predictor to 34 predictors plus age). In the second step, these 34 predictors plus age were fed to the machine learning model, which iteratively selected the next-most informative predictor to maximize performance during the training stage (34 predictors plus age to 15 predictors plus age).	No information on mortality<br><br>**16 predictors: Age, High nephrotoxic potential drugs, Vasoactive drugs, BUN, Total bilirubin, WBC, Serum chloride, Shock index, SpO2, SCr rate of change, PaCO2, AG, Serum albumin, Gentamicin trough, Mean airway pressure, Time since admission**<br>Stage 2/3 AKI: Prior to detection by conventional criteria with a median lead-time of 30 h at AUC of 0.89. The model predicted 70% of subsequent RRT episodes, 58% of Stage 2/3 episodes, and 41% of any AKI episodes. The ratio of false to true alerts of any AKI episodes was approximately 1:1 (PPV 47%).<br><br>Calibration plot: Calibrated risk curve close to diagonal；<br>AUC: IV, Hospital1=0.88, Hospital2=0.78, Hospital3=0.90.	—
**Wang et al, 2017**<br><br>**ICU patients**<br><br>**TRIPOD 3 – Derivation, IV & EV (28/37 pts)**	USA single-center retrospective cohort study (n=29263). Data from the Monroe Carell Jr. Children's Hospital at Vanderbilt admission between 1 January 2011 and 31 December 2012. Mean age with outcome 2.6 (0.6–10.5), without 6.5 (1.4–13.6) (P<0.001).<br>Included: derivation n=1332 & validation n=866.<br>Inclusion: age 28 days through 21 years, measurement of baseline and an additional inpatient SCr as defined below, and admission between 1 January 2011 and 31 December 2012.<br>Exclusion: Neonatal ICU admission (based on location codes) and CKD (ICD-9 and CPT codes).<br>Outcome: 1.5-fold or 0.3 mg/dl increase in SCr (KDIGO), without the restriction that the increase in Scr occurred within 48 h or 7 days. The baseline SCr was the lowest measurement obtained 90 days before through the first week of admission.<br>Predictor variables were selected from 29 candidate variables to balance the following goals: (1) real-time availability; (2) correlation to AKI; (3) limited collinearity; (iv) either low missingness or ability to transform to a categorical variable.<br>This studies internally validated and calibrated the models using bootstrapping technique.<br>Each model was assessed for overall performance (R2 and Brier Score), calibration (calibration plot, intercept and slope), and discrimination [Receiver operating characteristic (ROC) curve, Somer's D statistic, C-statistic, sensitivity and specificity]	No information on mortality.<br><br>Derivation AKI 791, Validations AKI 470.<br>**10 predictors: Age, High-risk nephrotoxins, Moderate risk nephrotoxins, Total medications, Platelet count, RDW, Phosphorus, Serum transaminases, pH, and Hypotension.**<br>Risk calculator supplied.<br><br>AUC: Derivation 0.75, IV 0.74, EV 0.74<br><br>Calibration plot: Derivation Calibration slope 1.00, IV 0.92, EV 0.94<br>Brier score: Derivation 0.20, IV 0.20, EV 0.20	Same study<br><br>AUC 0.74<br>Derivation<br>Calibration slope 0.94<br><br>Brier score 0.20.

Table S4. (Continue)

Critically ill patients in ICU

Author, Type, TRIPOD detail	Population, Outcome, AKI Definitions, Baseline Scr, Methods	Outcomes, Predictors & Model performance	External Validation
**Basu et al, 2014 TRIPOD 3,4 – Derivation, EV (27/37 pts)**	USA and Canada multi-center (19). Data from four separate PICU cohorts in CCHMC and MCH. C1 (n=144): derivation, CCHMC sepsis cohort1,2009-2010; C2–C4 (n=118, 108, and 214, respectively): validation, MCH prospective cohort, 2004-2007, MCH retrospective cohort, 2007-2010, CCHMC sepsis cohort 2, 2006-2011. Mean age 3.8 (1.2, 12.5), 3.0 (0.2, 11.7), 1.5 (0.3, 10.6) and 2.2 (0.8, 5.9), respectively. Male 83(57.6%), 74(62.7%), 64(59.2%) and 134(62.6%), respectively.<br>Included: derivation n=144 & validation n=118, 108, and 214, respectively.<br>Inclusion: C1, C4: admitted to the PICU with an International Classification of Diagnosis (ICD-9) code of 'sepsis' or 'septic shock', as per international consensus guidelines for the diagnosis of sepsis. C2, C3: All eligible children admitted to the MCH PICU for at least 4 days and 2 days, respectively.<br>Exclusion: Patients with preexisting chronic kidney disease or immediately after cardiac surgery were excluded (three patients were excluded from C1 because of baseline requirement of RRT).<br>Outcome: Stage2/3 AKI 72 h after PICU admission (KDIGO). A baseline SCr was also imputed based on the eCCl and the patient height using the Schwartz correction, or the lowest SCr up to 3 months before PICU admission	Mortality 13 (9.0%),7 (5.9%),4 (3.7%)，23 (10.7%), respectively in 4 cohorts.<br><br>Derivation Stage 2/3 AKI 28(19.4%), Validations Stage 2/3 AKI 12(10.2%), 11(10.2%) and 29(13.6%), respectively.<br><br>**Predictors: Renal angina index, combinations of biomarkers (SCr rate of change, Fluid overload, PICU admissions, History of solid organ or bone marrow transplant and intubated + presence of at least one vasopressor or inotrope)**<br><br>Derivation AUC 0.77 (0.68–0.86), EV AUC 0.74 (0.59–0.88), 0.81 (0.71–0.91), 0.80 (0.75–0.86)	Same Study different site<br><br>AUC<br>0.74 (0.59–0.88),<br>0.81 (0.71–0.91),<br>0.80 (0.75–0.86)
**Sanchez-Pinto et al, 2016**<br><br>**Critically Ill Children in general PICU**<br><br>**TRIPOD 3 – Derivation, IV & EV (30/37 pts)**	USA single-center retrospective cohort study (n=9,396) between May 2003 and March 2015. Data from the EHR clinical databases (Cerner Kids, Kansas City, MO; Philips/Care Vue, Waltham, MA) and a locally developed quality improvement and clinical database (Microsoft Access, Seattle, WA). Overall mean age 7.3 (1.7–13.6).<br>Inclusion (n=10475): All patients 1 month to 21 years old without AKI at admission and alive and in the ICU for at least 24 hours.<br>Exclusion (n=1079): Patients were excluded if they were younger than 1 month or older than 21 years, had AKI on admission, had documented CKD, were perioperative for a kidney transplant, or had no SCr levels measured.<br>Included (n=9396): derivation n=3932 & validation n=2632 and 2832, respectively.<br>Outcome: Early AKI, increase of SCr ＞1.5 times the baseline creatinine or an increase of at least 0.3mg/ dL in 48 hours (KDIGO staging criteria). A baseline SCr was the most recent creatinine obtained in the 3 months before admission (baseline SCr＜the low normal for age and gender, then baseline SCr= the low normal).<br>Missing values were assumed to be normal, and the normal category was assigned in those cases. Candidate variables within each of the nine pathophysiology groups in the derivation set were tested in multivariable logistic regression models against the other variables in their group. Those independently associated with Early AKI with a p value of less than or equal to 0.15 were selected for consideration in the final model.<br>Of the 33 candidate variables tested in each of the nine pathophysiologic groups, 17 had an association with early AKI of p≤0.15 in the group-level multivariable logistic regressions (Table 2). These 17 variables were tested in the final model. Ten of the 17 variables were eliminated by backward elimination (p > 0.15) in the final multivariable logistic regression model.	4% had early acute kidney injury, and these had significantly higher mortality than those without early acute kidney injury (26% vs 3.3%; p<0.001)<br><br>Derivation early AKI 161(4.1%); validation early AKI 102(3.9%) and 114(4%)<br><br>**7 predictors: Age, Postoperative recovery, Preadmission cardiac arrest, BUN, Platelet count, Total bilirubin, pH.**<br><br>Risk calculator supplied.<br><br>Derivation AUC 0.84 (0.83–0.86), IV AUC 0.81 (0.80–0.83) EV AUC 0.86 (0.85–0.88)	Same study<br><br>AUC<br>0.86 (0.85–0.88)

Table S4. **(Continue)**

Critically ill patients in ICU

Author, Type, TRIPOD detail	Population, Outcome, AKI Definitions, Baseline Scr, Methods	Outcomes, Predictors & Model performance	External Validation
**Sanchez-Pinto et al, 2018**	USA single-center observational cohort (n=6,564).	No information on mortality.	—
	Inclusion: Critically ill children admitted to a pediatric ICU without evidence of acute kidney injury. No exclusion criteria were specified.	Derivation AKI 157	
**Critically ill children admitted to a pediatric ICU**	Included (n=6564): derivation n=3938 & validation n =2626<br>Outcome: New AKI by 72 hours of ICU stay. A baseline SCr was the most recent creatinine obtained in the 3 months before admission (baseline SCr＜the low normal for age and gender, then baseline SCr= the low normal).<br>Missing values were imputed using multiple imputation methods and the original dataset was divided in a 60/40 split for derivation and validation, which resulted in 6 events per variable in the derivation.	**26 predictors: Age, pH, Weight, UOP, Bilirubin, BUN, Hemoglobin, Platelets, Potassium, WBC, Lowest SBP, SBP[SD], Lowest SF ratio, VIS, DIC score, on mechanical ventilation, male, black, cardiac arrest pre-admission, postoperative recovery, received vancomycin, received ampho B, received ganciclovir, received ACEI, received acyclovir, received ampicillin/tazobactam (Zosyn)**	
**TRIPOD 2A - Derivation, IV**	Eight variable selection methods: four regression-based methods (stepwise backward selection using p-value and AIC, Least Absolute Shrinkage and Selection Operator, and Elastic Net) and four tree-based methods (Variable Selection Using Random Forest, Regularized Random Forests, Boruta, and Gradient Boosted Feature Selection).	Internal validation AUC 0.837, 0.826, 0.832, 0.833, 0.809, 0.824, 0.817 and 0.785, respectively.	
**(15/37 pts)**	Method-specific modeling approaches: Regression-based methods & Tree-based methods.		

Patients in non-ICU

Author, Type, TRIPOD detail	Population, Outcome, AKI Definitions, Baseline Scr,, Methods	Outcomes, Predictors & Model performance	External Validation
**Wang et al, 2017**	USA single-center retrospective cohort study(n=29263). Data from the Monroe Carell Jr. Children's Hospital at Vanderbilt admission between 1 January 2011 and 31 December 2012. Mean age with outcome 2.6 (0.6–10.5), without 6.5 (1.4–13.6) (P<0.001).	No information on mortality.<br>Derivation AKI 722, Validations AKI 469.	Same study
**TRIPOD 3 – Derivation, IV & EV**<br>**(28/37 pts)**	Included: derivation n=2337 & validation n=1474.<br>Inclusion: age 28 days through 21 years, measurement of baseline and an additional inpatient Scr as defined below, and admission between 1 January 2011 and 31 December 2012.<br>Exclusion: Neonatal ICU admission (based on location codes) and chronic kidney disease (ICD-9 and CPT codes). Derivation n=1311 & validation n=321	**8 predictors: Age, High-risk nephrotoxins, Moderate risk nephrotoxins, Total medications, Platelet count, Median RDW, Phosphorus, Serum transaminases.**	Derivation<br>Calibration slope 0.95
	Outcome: 1.5-fold or 0.3 mg/dl increase in Scr (KDIGO), without the restriction that the increase in Scr occurred within 48 h or 7 days. The baseline SCr was the lowest measurement obtained 90 days before through the first week of admission.	Risk calculator supplied.	Brier score 0.20
	Predictor variables were selected from 29 candidate variables to balance the following goals: (1) real-time availability; (2) correlation to AKI; (3) limited collinearity; (4) either low missingness or ability to transform to a categorical variable.<br>This studies internally validated and calibrated the models using bootstrapping technique.<br>Each model was assessed for overall performance (R2 and Brier Score), calibration (calibration plot, intercept and slope), and discrimination [Receiver operating characteristic (ROC) curve, Somer's D statistic, C-statistic, sensitivity and specificity]	Derivation AUC 0.69, IV 0.69, EV 0.69.<br>Calibration plot: Derivation Calibration slope 1.00, IV 0.93, EV 0.95<br>Brier score: Derivation 0.19, IV 0.19, EV 0.20	AUC 0.69

Table S4. **(Continue)**

Septic Shock

**Author, Type, TRIPOD detail**	**Population, Outcome, AKI Definitions, Baseline Scr, Methods**	**Outcomes, Predictors & Model performance**	**External Validation**
**Stanski et al, 2020**	USA multi-center (14) prospective cohort (n=379) from January 2015 to December 2018 (27). Mean age with outcome 5.1 (1.3–13.5), without outcome 6.5 (2.5–12.4). Male with outcome 33 (51%), without outcome 162 (52%).	28-day Mortality with outcome 24 (37%), without outcome 18 (6%).	**Stenson EK, et al. 2018**
**Septic Shock**	Inclusion (n=461): Children between the ages of 1 week and 18 years admitted with septic shock to PICUs. The inclusion criteria were based on pediatric-specific consensus criteria (33).	379 patients, 65 (17%) developed severe D3 SA-AKI	
**TRIPOD 1B,4 Derivation, IV (28/37 pts)**	Exclusion (n=82): The inability to obtain informed consent. We excluded patients without any SCr data, those still admitted to the PICU on Day 3 of septic shock but with missing Day 3 SCr values, those discharged from the PICU alive before Day 3 with evidence of AKI, and those with preexisting kidney disease (n=82).	**5 predictors: Day 1 KDIGO AKI stage, PERSEVERE-II mortality probability and the PERSEVERE biomarkers (IL-8, HSPA1B, GZMB).**	In the historical test cohort (n = 461)
	Included (n=379): patients who had SCr data vailable from both admission (Day 1) and Day 3 of septic shock (n=328)，patients who had been discharged from the PICU alive before Day 3 and without evidence of AKI (n=51).	The AUC of the decision tree for discriminating between patients with and without severe D3 SA-AKI was 0.95 (95% CI, 0.92–0.98).	The decision tree AUC 0.83 (0.79–0.88)
	Outcome: Severe D3 SA-AKI. Severe SA-AKI was defined as KDIGO stage 2 AKI or higher, which is at least a twofold increase in SCr from baseline. RRT use and of renal recovery by Day 3 of septic shock. Renal recovery was defined as any improvement of AKI stage by Day 3 among patients with early AKI, which was defined as KDIGO stage 1 or higher on Day 1 or 2 of septic shock. Baseline SCr was estimated for each patient using calculated body surface area (in square meters) and an estimated glomerular filtration rate of 120 mL/min per 1.73 m^2, as validated in the literature. When patient height data were unavailable (n = 20), the age-based Pottel method was used.	On 10-fold cross-validation, the decision tree had a summary AUC of 0.88.	P＜0.001
	Performance of the derived model is reported using diagnostic test statistics and was tested using a 10-fold cross-validation procedure.		

Chronic Liver Diseases

**Author, Type, TRIPOD detail**	**Population, Outcome, AKI Definitions, Baseline Scr, Methods**	**Outcomes, Predictors & Model performance**	**External Validation**
**Vijay et al, 2020**	India single-center Prospective observational study (n=247).	247 children with CLD, 41(16.5%) developed AKI. Mean age at AKI was 8 ± 5.4 years. 16 patients with AKI died.	—
**Chronic Liver Diseases**	All patients of CLD with PHTN with or without AKI were enrolled and followed up for 3 months for development/resolution of AKI as per KDIGO definition. No information on the definition of the baseline SCr.	AKI score: -7.34 +6.3 (Cystatin in mg/L) + 0.006 (Urinary NGAL in ng/ml)]	
**TRIPOD 1A - Derivation (28/37 pts)**	AKI score was calculated using newer biomarkers viz. cystatin C, urinary NGAL and RRI	Derivation AUC 0.926	

Table S4. **(Continue)**

Very Low Birth Weight Infants

Author, Type, TRIPOD detail	Population, Outcome, AKI Definitions, Baseline Scr, Methods	Outcomes, Predictors & Model performance	External Validation
**Hu et al, 2020**	China single-center prospective cohort (n=604). Children's Hospital of Chongqing Medical University between January 2012 and October 2018. Overall mean Age(h) 604, mean Age(h) with and without outcome 144,460. Overall female 299(49.5%), female with and without outcome 61(42.4%), 238(51.7%).	604 VLBW infants, of which 144 (23.8%) developed AKI; in 111 (77.1%) of these infants, AKI occurred within 7 days of birth.	—
**TRIPOD 1B - Derivation, IV (26/37 pts)**	Inclusion (n=926): age of ≤3 days and birth weight of <1,500 g.<br>Exclusion (n=322): (1) severe congenital malformation or inherited metabolic diseases, (2) maternal history of kidney diseases or abnormal renal function, (3) the infant died or was discharged within 3 days of admission, and (4) <2 measurements of SCr levels of the infant during hospitalization.	**6 predictors: GA, maternal age of≥35 years, low RBC count within 3 days of birth, low Ca concentration within 3 days of birth, and history of PAH or myocardial injury**	
	Included (n=604): patients who had SCr data vailable from both admission (Day 1) and Day 3 of septic shock (n = 328), patients who had been discharged from the PICU alive before Day 3 and without evidence of AKI (n = 51).	Derivation AUC 0.794 (0.754–0.834), IV AUC 0.788	
	Outcome: 1/2/3stage AKI occurs within three days after birth (KDIGO). The baseline SCr level for each infant was defined as the lowest level recorded previously since the baseline level changes constantly during the first week of birth.	The Hosmer–Lemeshow test (P = 0.245)	
	Forward stepwise regression analysis was performed to screen for independent variables. The Hosmer–Lemeshow test was used to evaluate the goodness of fit of the model.<br>The model was internally validated using the 10-fold cross-validation method. A nomogram was plotted to predict the risk of AKI in VLBW infants on the basis of the results of multivariate logistic regression analysis.		

Abbreviation: Acute kidney injury, AKI; Renal replacement therapy, RRT; Electronic health records, EHRs; external validation, EV; internal validation, IV; Chronic Liver Diseases, CLD; Kidney Disease: Pulmonary Hypertension , Newborn, PHTN; Improving Global Outcomes, KDIGO; Cincinnati Children's Hospital Medical Center, CCHMC; Montreal Children's Hospital, MCH; Cardiothoracic intensive care units, CTICU; Internal Validation, IV; External Validation, EV; chronic kidney disease, CKD; the area under the receiver operating characteristic, AUC; Serum creatinine, SCr; Angiotensin-Converting Enzyme Inhibitors, ACEI; Amphotericin B, Ampho B; Blood Urea Nitrogen, BUN; White Blood Cell Count, WBC; Lowest SaO2/FiO2 (SF) ratio, Lowest SF ratio; Urine output, UOP; Systolic blood pressure, SBP; Red blood cell distribution width, RDW; Heat shock protein 70 kD 1B, HSPA1B; Anion Gap, AG; Red Blood Cells Count, RBC; Disseminated intravascular coagulopathy score, DIC score; Vasoactive-inotropic score, VIS; granzyme B, GZMB; Renal resistive index, RRI.

Table S5. Limitations in methodology and reporting

Area of concern	Description
Missing data	Multiple imputation was a recommended method of dealing with missing value to avoid bias, however it was described in only three studies.
Definitions of outcome and predictors	Three studies excluded patients with pre-existing CKD; three studies took the lowest SCr up to three months before PICU admission as baseline, two studies took the most recent SCr, two studies took the mean normal creatinine level for age and gender group. Two studies included the change of SCr as predictor despite it forming the outcome; the time span used to define the outcome is unclear in two studies.
Blinding of predictors or outcome	All studies did not report blinding methods for their respective collecting predictors or outcomes, so that information bias in research is unavoidable.
Sample size	Calculations of sample size were not described in all studies, eleven models had EPP <10. Small sample increases risk of overfitting and underfitting.
Univariate to select for multivariate analysis	It is not recommended to select variables only based on the univariate analyses, however it was used in thirteen of sixteen models.
The handling of variables with age-dependent trends	Three studies used age-normalized to address predictors with age-dependent trends, which may cause the loss of original information. Only one study used age-dependent ensemble machine learning model.
Modelling methods	All studies did not report new modelling techniques such as RNN model, six of eight studies used logistic regression.
Calibration plots	Important part of model performance, present in only three model and two external validations.
External validation and model updating	Few have external validations, updating is recommended but not described.
Newer performance measures	Techniques such as decision curve analysis can offer insight into clinical consequences, but it was not described in all studies.
Electronic automation	Incomplete reporting and little consideration of electronic automation influenced the wide use.

Abbreviation: CKD, chronic kidney disease; RNN, recurrent neural network.

Table S6. TRIPOD items reported in the 8 studies

Section	Topic	items	Dong et al, 2021	Wang et al, 2017	Basu et al, 2014	Sanchez et al, 2016	Sanchez et al, 2018	Vijay et al, 2020	Stanskji et al, 2020	Hu et al, 2020	Total (/8)
Title and abstract	Title	1	1	1	1	1	1	1	1	1	8
	Abstract	2	1	1	1	1	1	1	1	1	8
Introduction	Background and objectives	3a	1	1	1	1	1	1	1	1	8
		3b	1	1	1	1	0	1	1	1	7
Methods	Source of data	4a	1	1	1	1	0	0	1	1	6
		4b	1	1	1	1	0	0	1	1	6
	Participants	5a	1	1	1	1	0	0	1	1	6
		5b	1	1	1	1	1	1	0	0	6
		5c	1	0	0	0	0	0	1	1	3
	Outcome	6a	1	1	1	1	1	1	0	0	6
		6b	0	0	0	0	0	0	1	1	2
	Predictors	7a	0	0	1	1	0	1	1	0	4
		7b	0	0	0	0	0	0	0	0	0
	Sample size	8	0	0	0	1	0	0	0	0	1
	Missing data	9	0	1	0	0	1	0	0	1	3
	Statistical analysis methods	10a	1	1	1	1	1	1	1	1	8
		10b	1	1	1	1	1	1	1	1	8
		10c	1	1	1	1	0	1	1	0	6
		10d	0	0	1	0	1	0	1	0	3
		10e	0	0	0	0	0	0	0	0	0
	Risk groups	11	0	0	1	1	0	0	1	0	3
	Development vs. validation	12	0	1	1	1	0	0	0	1	4
Results	Participants	13a	1	1	0	1	0	1	1	1	6
		13b	0	1	0	1	0	0	1	1	4
		13c	0	1	1	1	0	0	0	0	3
	Model development	14a	0	1	1	1	0	1	1	1	6
		14b	0	1	0	1	0	1	1	1	5
	Model specification	15a	0	1	1	1	0	1	1	1	6
		15b	1	1	1	1	1	0	1	1	7
	Model performance	16	1	1	1	1	0	1	1	1	7
	Model updating	17	0	0	0	0	0	0	0	0	0
Discussion	Limitations	18	1	1	1	1	1	0	1	1	7
	Interpretation	19a	1	1	1	1	1	0	1	1	7
		19b	1	1	1	1	1	0	1	1	7
	Implications	20	1	1	1	1	1	0	1	1	7
Other information	information	21	1	1	1	1	0	0	1	1	6
	Funding	22	1	1	1	1	1	1	1	1	8
	Score*		22	28	27	30	15	16	28	26	

*A global TRIPOD score for each study was calculated to quantify reporting, consisting of the sum of the scores for each individual item (Out of a maximum of 37, with a score of 1 if the was criterion met, and a score of 0 for each item that did not meet the criteria, or if it was uncle).

Table S7. Risk of bias evaluation rules based on Prediction study Risk Of Bias Assessment Tool (PROBAST)

Author, Year [Reference]	ROB																							Applicability			Overall	
	Participants			Predictors				Outcome							Analysis									Participants	Predictors	Outcome	ROB	Applicability
	Overall	1.1	1.2	Overall	2.1	2.2	2.3	Overall	3.1	3.2	3.3	3.4	3.5	3.6	Overall	4.1	4.2	4.3	4.4	4.5	4.6	4.7	4.8	4.9					
Dong et al， 2021	?	Y	pN	-	pN	pN	N	-	Y	Y	N	Y	Y	Y	-	N	N	Y	Y	Y	pN	Y	Y	Y	+	+	-	-	-
Wang et al， 2017-1	?	Y	pN	-	Y	pN	N	?	Y	Y	Y	Y	Y	pN	-	Y	Y	N	N	pN	pN	Y	Y	Y	+	+	+	-	+
Basu et al， 2014	?	Y	pN	?	pN	pN	Y	-	Y	Y	N	Y	Y	Y	-	N	Y	pN	pN	N	pN	Y	pN	Y	+	+	-	-	-
Sanchez et al， 2016	?	Y	pN	?	Y	pN	Y	+	Y	Y	Y	Y	Y	Y	-	N	Y	pN	pN	Y	pN	Y	Y	Y	+	+	+	-	+
Sanchez et al， 2018-1	?	Y	pN	-	Y	pN	N	?	Y	Y	Y	Y	Y	pN	-	N	Y	Y	Y	Y	pN	pN	N	pN	+	+	+	-	+
Sanchez et al， 2018-2	?	Y	pN	-	Y	pN	N	?	Y	Y	Y	Y	Y	pN	-	N	Y	Y	Y	Y	pN	pN	N	pN	+	+	+	-	+
Sanchez et al， 2018-3	?	Y	pN	-	Y	pN	N	?	Y	Y	Y	Y	Y	pN	-	N	Y	Y	Y	Y	pN	pN	N	pN	+	+	+	-	+
Sanchez et al， 2018-4	?	Y	pN	-	Y	pN	N	?	Y	Y	Y	Y	Y	pN	-	N	Y	Y	Y	Y	pN	pN	N	pN	+	+	+	-	+
Sanchez et al， 2018-5	?	Y	pN	-	Y	pN	N	?	Y	Y	Y	Y	Y	pN	-	N	Y	Y	Y	pN	pN	pN	N	pN	+	+	+	-	+
Sanchez et al， 2018-6	?	Y	pN	-	Y	pN	N	?	Y	Y	Y	Y	Y	pN	-	N	Y	Y	Y	pN	pN	pN	N	pN	+	+	+	-	+
Sanchez et al， 2018-7	?	Y	pN	-	Y	pN	N	?	Y	Y	Y	Y	Y	pN	-	N	Y	Y	Y	pN	pN	pN	N	pN	+	+	+	-	+
Sanchez et al， 2018-8	?	Y	pN	-	Y	pN	N	?	Y	Y	Y	Y	Y	pN	-	N	Y	Y	Y	pN	pN	pN	N	pN	+	+	+	-	+
Wang et al， 2017-2	?	Y	pN	-	Y	pN	N	?	Y	Y	Y	Y	Y	pN	-	Y	Y	N	N	pN	pN	Y	Y	Y	+	+	+	-	+
Vijay et al， 2020	?	pN	pN	-	Y	pN	N	?	Y	Y	Y	Y	Y	pN	-	N	pN	pN	pN	Y	pN	pN	N	Y	?	+	+	-	?
Stanskji et al， 2020	?	Y	pN	-	Y	pN	N	?	Y	Y	Y	Y	Y	pN	-	N	pN	pN	pN	Y	pN	Y	Y	Y	?	?	?	-	?
Hu et al， 2020	?	Y	pN	?	Y	pN	Y	?	Y	Y	Y	Y	Y	pN	-	N	pN	pN	pN	Y	pN	Y	Y	Y	?	?	+	-	?

PROBAST = Prediction Model Risk of Bias Assessment Tool; ROB = risk of bias; N=No; Y=Yes; pN= unclear. * + indicates low ROB/low concern regarding applicability; − indicates high ROB/high concern regarding applicability; ? indicates unclear ROB/unclear concern regarding applicability

PROBAST consists of 4 domains containing 20 signaling questions to facilitate ROB assessment and 3 domains containing 3 signaling questions to performed Applicability assessment:

Participants Domians:

Risk of Bias: 1.1 Were appropriate data sources used, e.g. cohort, RCT or nested case-control study data? 1.2 Were all inclusions and exclusions of participants appropriate?

Applicability: Concern that the included participants and setting do not match the review question

Predictors Domians:

Risk of Bias: 2.1 Were predictors defined and assessed in a similar way for all participants? 2.2 Were predictor assessments made without knowledge of outcome data? 2.3 Are all predictors available at the time the model is intended to be used?

Applicability: Concern that the definition, assessment or timing of predictors in the model do not match the review question

Outcome Domians:

Risk of Bias: 3.1 Was the outcome determined appropriately? 3.2 Was a pre-specified or standard outcome definition used? 3.3 Were predictors excluded from the outcome definition? 3.4 Was the outcome defined and determined in a similar way for all participants? 3.5 Was the outcome determined without knowledge of predictor information? 3.6 Was the time interval between predictor assessment and outcome determination appropriate?

Applicability: Concern that the outcome, its definition, timing or determination do not match the review question

Analysis Domians:

Risk of Bias: 4.1 Were there a reasonable number of participants with the outcome? 4.2 Were continuous and categorical predictors handled appropriately? 4.3 Were all enrolled participants included in the analysis? 4.4 Were participants with missing data handled appropriately? 4.5 Was selection of predictors based on univariable analysis avoided? 4.6 Were complexities in the data (e.g. censoring, competing risks, sampling of controls) accounted for appropriately? 4.7 Were relevant model performance measures evaluated appropriately? 4.8 Were model overfitting and optimism in model performance accounted for? 4.9 Do predictors and their assigned weights in the final model correspond to the results from multivariable analysis?

Table S8. Most common predictors used in the 16 models

Field	Critically ill Pediatric patients in ICU												No-ICU	Chronic Liver Diseases	Septic Shock	Very Low Birth Weight Infants	Total
**Author, year**	**Dong, 2021**	**Wang, 2017-1**	**Basu, 2014**	**Sanchez ,2016**	**Sanchez , 2018-1**	**Sanchez, 2018-2**	**Sanchez ,2018-3**	**Sanchez ,2018-4**	**Sanchez ,2018-5**	**Sanchez ,2018-6**	**Sanchez, 2018-7**	**Sanchez ,2018-8**	**Wang, 2017-2**	**Vijay, 2018**	**Stanski, 2022**	**Hu, 2020**	
**Demographics**																	
Weight						×	×	×	×	×	×	×					7
Age	×	×		×									×				4
**Medications**																	
Ampicillin/tazobactam (Zosyn)					×	×	×	×		×	×						6
Acyclovir						×	×	×			×	×					5
ACEI					×	×	×	×		×							5
High nephrotoxic potential drugs	×	×											×				3
**Labs**																	
PLT		×		×	×	×	×	×	×	×	×	×	×				11
BUN	×			×	×	×	×	×	×	×	×	×					10
pH		×		×	×	×	×	×	×	×	×	×					10
TBil	×			×		×	×	×	×	×	×	×					9
WBC	×						×	×		×	×						5
Hemoglobin							×	×	×	×	×						5
Potassium						×	×	×		×	×						5
Lowest SF ratio							×	×		×	×	×					5
UOP					×		×	×			×						4
SPB							×	×			×						3
**Other**																	
Postoperative recovery				×	×	×	×	×	×	×	×						8
DIC score					×	×	×	×	×	×	×	×					8
Preadmission cardiac arrest				×	×	×	×	×		×		×					7
VIS							×	×		×	×						4

Abbreviation: Angiotensin-Converting Enzyme Inhibitors, ACEI; Platelet count, PLT; Blood Urea Nitrogen, BUN; Total bilirubin, TBil; White Blood Cell Count, WBC; Lowest SaO2/FiO2 (SF) ratio, Lowest SF ratio; Urine output, UOP; Systolic blood pressure, SPB; Disseminated intravascular coagulopathy score, DIC score; Vasoactive-inotropic score, VIS.

Table S9. All predictors included in the 16 models

Field	Critically ill Pediatric patients in ICU												No-ICU	Chronic Liver Diseases	Septic Shock	Very Low Birth Weight Infants	Total
Author, year	Dong, 2021	Wang, 2017-1	Basu, 2014	Sanchez ,2016	Sanchez , 2018-1	Sanchez, 2018-2	Sanchez ,2018-3	Sanchez ,2018-4	Sanchez ,2018-5	Sanchez ,2018-6	Sanchez, 2018-7	Sanchez ,2018-8	Wang, 2017-2	Vijay, 2018	Stanski, 2022	Hu, 2020	
**Demographics**																	
Weight						×	×	×	×	×	×	×					7
Age	×	×		×									×				4
Gender (Male)											×						1
Gestational age																×	1
Maternal age																×	1
**Medications**																	
Ampicillin/tazobactam (Zosyn)					×	×	×	×		×	×						6
Acyclovir						×	×	×			×	×					5
ACEI					×	×	×	×		×							5
High nephrotoxic potential drugs	×	×											×				3
Moderate risk nephrotoxins		×											×				2
Total medications		×											×				2
Ganciclovir							×	×									2
Ampho B							×	×									2
Vancomycin											×						1
Vasoactive drugs	×																1
**Diagnoses**																	
**Day 1 KDIGO AKI stage**															×		1
Hypotension		×															1
Pulmonary hypertension																×	1
Myocardial injury																×	1
**Labs**																	
PLT		×		×	×	×	×	×	×	×	×	×	×				11
BUN	×			×	×	×	×	×	×	×	×	×					10
pH		×		×	×	×	×	×	×	×	×	×					10
TBil	×			×		×	×	×	×	×	×	×					9
WBC	×						×	×		×	×						5
Hemoglobin							×	×	×	×	×						5
Potassium						×	×	×		×	×						5
Lowest SF ratio							×	×		×	×	×					5
UOP					×		×	×			×						4
SPB							×	×			×						3
Serum chloride	×																2
RDW		×											×				2
Serum phosphorus		×											×				2
Serum transaminases		×											×				2

Abbreviation: Angiotensin-Converting Enzyme Inhibitors, ACEI; Amphotericin B, Ampho B; Serum creatinine, SCr; Platelet count, PLT; Blood Urea Nitrogen, BUN; Total bilirubin, TBil; White Blood Cell Count, WBC; Lowest SaO2/FiO2 (SF) ratio, Lowest SF ratio; Urine output, UOP; Systolic blood pressure, SBP; Red blood cell distribution width, RDW; Heat shock protein 70 kD 1B, HSPA1B; Anion Gap, AG; Red Blood Cells Count, RBC; Disseminated intravascular coagulopathy score, DIC score; Vasoactive-inotropic score, VIS; PERSEVERE-II mortality probability, PERSEVERE-II; granzyme B, GZMB; History of solid organ or bone marrow transplant, HSO&HBMT; Intubated + presence of at least one vasopressor or inotrope, Intubated + vasopressor or inotrope; Renal resistive index, RRI.

Table S9. (Continue)

Field	Critically ill Pediatric patients in ICU												No-ICU	Chronic Liver Diseases	Septic Shock	Very Low Birth Weight Infants	Total
Author, year	Dong, 2021	Wang, 2017-1	Basu, 2014	Sanchez ,2016	Sanchez , 2018-1	Sanchez, 2018-2	Sanchez ,2018-3	Sanchez ,2018-4	Sanchez ,2018-5	Sanchez ,2018-6	Sanchez, 2018-7	Sanchez ,2018-8	Wang, 2017-2	Vijay, 2018	Stanski, 2022	Hu, 2020	
**Labs**																	
HSPA1B															×		1
IL-8															×		1
Shock index	×																1
SpO2	×																1
Scr rate of change	×		×														1
PaCO2	×																1
AG	×																1
Serum albumin	×																1
Gentamicin trough	×																1
Fluid overload			×														1
RBC																×	1
Ca																×	1
Cystatin C														×			1
Urinary NGAL														×			1
**Other**																	
Postoperative recovery				×	×	×	×	×	×	×	×						8
DIC score					×	×	×	×	×	×	×	×					8
Preadmission cardiac arrest				×	×	×	×	×		×		×					7
VIS score							×	×		×	×						4
Mechanical ventilation Ventilator							×	×									2
Mean airway pressure	×																1
PERSEVERE-II															×		1
Time since admission	×																1
PICU admissions			×														1
GZMB															×		1
HSO&HBMT			×														1
Intubated + vasopressor or inotrope			×														1
RRI														×			1

Abbreviation: Angiotensin-Converting Enzyme Inhibitors, ACEI; Amphotericin B, Ampho B; Serum creatinine, SCr; Platelet count, PLT; Blood Urea Nitrogen, BUN; Total bilirubin, TBil; White Blood Cell Count, WBC; Lowest SaO2/FiO2 (SF) ratio, Lowest SF ratio; Urine output, UOP; Systolic blood pressure, SBP; Red blood cell distribution width, RDW; Heat shock protein 70 kD 1B, HSPA1B; Anion Gap, AG; Red Blood Cells Count, RBC; Disseminated intravascular coagulopathy score, DIC score; Vasoactive-inotropic score, VIS; PERSEVERE-II mortality probability, PERSEVERE-II; granzyme B, GZMB; History of solid organ or bone marrow transplant, HSO&HBMT; Intubated + presence of at least one vasopressor or inotrope, Intubated + vasopressor or inotrope; Renal resistive index, RRI.

Field	Critically ill Pediatric patients in ICU					No-ICU	CLD	Septic Shock	VLBW Infants	Total
Author, year	Dong, 2021	Wang, 2017	Basu, 2014	Sanchez, 2016	Sanchez, 2018	Wang, 2017	Vijay, 2018	Stanski, 2022	Hu, 2020	
**Demographics**										
Age	×	×		×	×	×				5
Weight					×					1
Gender (Male)					×					1
Gestational age									×	1
Maternal age									×	1
**Medications**										
High nephrotoxic potential drugs	×	×				×				3
Moderate risk nephrotoxins		×				×				2
Total medications		×				×				2
Ampicillin/tazobactam (Zosyn)					×					1
Acyclovir					×					1
ACEI					×					1
Ganciclovir					×					1
Ampho B					×					1
Vancomycin					×					1
Vasoactive drugs	×									1
**Diagnoses**										0
**Day 1 KDIGO AKI stag**e								×		1
Hypotension		×								1
Pulmonary hypertension									×	1
Myocardial injury									×	1
**Labs**										0
PLT		×		×	×	×				4
BUN	×			×	×					3
pH		×		×	×					3
TBil	×			×	×					3
**Scr rate of change**	×		×							2
WBC	×				×					2
RDW		×				×				2
Serum phosphorus		×				×				2
Serum transaminases		×				×				2
Hemoglobin					×					1
Potassium					×					1
Lowest SF ratio					×					1
UOP					×					1
SPB					×					1
Serum chloride	×									1
HSPA1B								×		1
IL-8								×		1
Shock index	×									1
SpO2	×									1
PaCO2	×									1
AG	×									1
Serum albumin	×									1
Gentamicin trough	×									1
Fluid overload			×							1
RBC									×	1
Ca									×	1
Cystatin C							×			1
Urinary NGAL							×			1

Table S10. All predictors included in the 8 studies

Abbreviation: Angiotensin-Converting Enzyme Inhibitors, ACEI; Amphotericin B, Ampho B; Serum creatinine, SCr; Platelet count, PLT; Blood Urea Nitrogen, BUN; Total bilirubin, TBil; White Blood Cell Count, WBC; Lowest SaO2/FiO2 (SF) ratio, Lowest SF ratio; Urine output, UOP; Systolic blood pressure, SBP; Red blood cell distribution width, RDW; Heat shock protein 70 kD 1B, HSPA1B; Anion Gap, AG; Red Blood Cells Count, RBC; Disseminated intravascular coagulopathy score, DIC score; Vasoactive-inotropic score, VIS; PERSEVERE-II mortality probability, PERSEVERE-II; granzyme B, GZMB; History of solid organ or bone marrow transplant, HSO&HBMT; Intubated + presence of at least one vasopressor or inotrope, Intubated + vasopressor or inotrope; Renal resistive index, RRI.

Table S10. (Continue)

Field	Critically ill Pediatric patients in ICU					No-ICU	CLD	Septic Shock	VLBW Infants	Total
**Author, year**	**Dong, 2021**	**Wang, 2017**	**Basu, 2014**	**Sanchez, 2016**	**Sanchez, 2018**	**Wang, 2017**	**Vijay, 2018**	**Stanski, 2022**	**Hu, 2020**	
**Other**										
Postoperative recovery				×	×					2
Preadmission cardiac arrest				×	×					2
DIC score					×					1
VIS score					×					1
Mechanical ventilation Ventilator					×					1
Mean airway pressure	×									1
PERSEVERE-II								×		1
Time since admission	×									1
PICU admissions			×							1
GZMB								×		1
HSO&HBMT			×							1
Intubated + vasopressor or inotrope			×							1
RRI							×			1

Abbreviation: Angiotensin-Converting Enzyme Inhibitors, ACEI; Amphotericin B, Ampho B; Serum creatinine, SCr; Platelet count, PLT; Blood Urea Nitrogen, BUN; Total bilirubin, TBil; White Blood Cell Count, WBC; Lowest SaO2/FiO2 (SF) ratio, Lowest SF ratio; Urine output, UOP; Systolic blood pressure, SBP; Red blood cell distribution width, RDW; Heat shock protein 70 kD 1B, HSPA1B; Anion Gap, AG; Red Blood Cells Count, RBC; Disseminated intravascular coagulopathy score, DIC score; Vasoactive-inotropic score, VIS; PERSEVERE-II mortality probability, PERSEVERE-II; granzyme B, GZMB; History of solid organ or bone marrow transplant, HSO&HBMT; Intubated + presence of at least one vasopressor or inotrope, Intubated + vasopressor or inotrope; Renal resistive index, RRI.

Table S11. PRISMA 2020 Checklist

Section and Topic	Item #	Checklist item	Location where item is reported
**TITLE**			
Title	1	Identify the report as a systematic review.	√1
**ABSTRACT**			
Abstract	2	See the PRISMA 2020 for Abstracts checklist.	√4
**INTRODUCTION**			
Rationale	3	Describe the rationale for the review in the context of existing knowledge.	√6-7
Objectives	4	Provide an explicit statement of the objective(s) or question(s) the review addresses.	√7
**METHODS**			
Eligibility criteria	5	Specify the inclusion and exclusion criteria for the review and how studies were grouped for the syntheses.	√7-8
Information sources	6	Specify all databases, registers, websites, organisations, reference lists and other sources searched or consulted to identify studies. Specify the date when each source was last searched or consulted.	√7-8
Search strategy	7	Present the full search strategies for all databases, registers and websites, including any filters and limits used.	√8
Selection process	8	Specify the methods used to decide whether a study met the inclusion criteria of the review, including how many reviewers screened each record and each report retrieved, whether they worked independently, and if applicable, details of automation tools used in the process.	√8
Data collection process	9	Specify the methods used to collect data from reports, including how many reviewers collected data from each report, whether they worked independently, any processes for obtaining or confirming data from study investigators, and if applicable, details of automation tools used in the process.	√8
Data items	10a	List and define all outcomes for which data were sought. Specify whether all results that were compatible with each outcome domain in each study were sought (e.g., for all measures, time points, analyses), and if not, the methods used to decide which results to collect.	√9
	10b	List and define all other variables for which data were sought (e.g., participant and intervention characteristics, funding sources). Describe any assumptions made about any missing or unclear information.	/
Study risk of bias assessment	11	Specify the methods used to assess risk of bias in the included studies, including details of the tool(s) used, how many reviewers assessed each study and whether they worked independently, and if applicable, details of automation tools used in the process.	√8-9
Effect measures	12	Specify for each outcome the effect measure(s) (e.g., risk ratio, mean difference) used in the synthesis or presentation of results.	/
Synthesis methods	13a	Describe the processes used to decide which studies were eligible for each synthesis (e.g., tabulating the study intervention characteristics and comparing against the planned groups for each synthesis (item #5)).	/
	13b	Describe any methods required to prepare the data for presentation or synthesis, such as handling of missing summary statistics, or data conversions.	/
	13c	Describe any methods used to tabulate or visually display results of individual studies and syntheses.	/
	13d	Describe any methods used to synthesize results and provide a rationale for the choice(s). If meta-analysis was performed, describe the model(s), method(s) to identify the presence and extent of statistical heterogeneity, and software package(s) used.	/
	13e	Describe any methods used to explore possible causes of heterogeneity among study results (e.g., subgroup analysis, meta-regression).	/
	13f	Describe any sensitivity analyses conducted to assess robustness of the synthesized results.	/
Reporting bias assessment	14	Describe any methods used to assess risk of bias due to missing results in a synthesis (arising from reporting biases).	/
Certainty assessment	15	Describe any methods used to assess certainty (or confidence) in the body of evidence for an outcome.	/
**RESULTS**			
Study selection	16a	Describe the results of the search and selection process, from the number of records identified in the search to the number of studies included in the review, ideally using a flow diagram.	√10
	16b	Cite studies that might appear to meet the inclusion criteria, but which were excluded, and explain why they were excluded.	√10
Study characteristics	17	Cite each included study and present its characteristics.	√10
Risk of bias in studies	18	Present assessments of risk of bias for each included study.	√11
Results of individual studies	19	For all outcomes, present, for each study: (a) summary statistics for each group (where appropriate) and (b) an effect estimation and its precision (e.g., confidence/credible interval), ideally using structured tables or plots.	√11-12
Results of syntheses	20a	For each synthesis, briefly summarise the characteristics and risk of bias among contributing studies.	/
	20b	Present results of all statistical syntheses conducted. If meta-analysis was done, present for each the summary estimate and its	/

		precision (e.g., confidence/credible interval) and measures of statistical heterogeneity. If comparing groups, describe the direction of the effect.	
	20c	Present results of all investigations of possible causes of heterogeneity among study results.	/
	20d	Present results of all sensitivity analyses conducted to assess the robustness of the synthesized results.	/
Reporting biases	21	Present assessments of risk of bias due to missing results (arising from reporting biases) for each synthesis assessed.	√11
Certainty of evidence	22	Present assessments of certainty (or confidence) in the body of evidence for each outcome assessed.	/
**DISCUSSION**			
Discussion	23a	Provide a general interpretation of the results in the context of other evidence.	√13-16
	23b	Discuss any limitations of the evidence included in the review.	√16-17
	23c	Discuss any limitations of the review processes used.	√16-17
	23d	Discuss implications of the results for practice, policy, and future research.	√17-18
**OTHER INFORMATION**			
Registration and protocol	24a	Provide registration information for the review, including register name and registration number, or state that the review was not registered.	√20
	24b	Indicate where the review protocol can be accessed, or state that a protocol was not prepared.	√20
	24c	Describe and explain any amendments to information provided at registration or in the protocol.	/
Support	25	Describe sources of financial or non-financial support for the review, and the role of the funders or sponsors in the review.	√19
Competing interests	26	Declare any competing interests of review authors.	√20
Availability of data, code and other materials	27	Report which of the following are publicly available and where they can be found: template data collection forms; data extracted from included studies; data used for all analyses; analytic code; any other materials used in the review.	√19

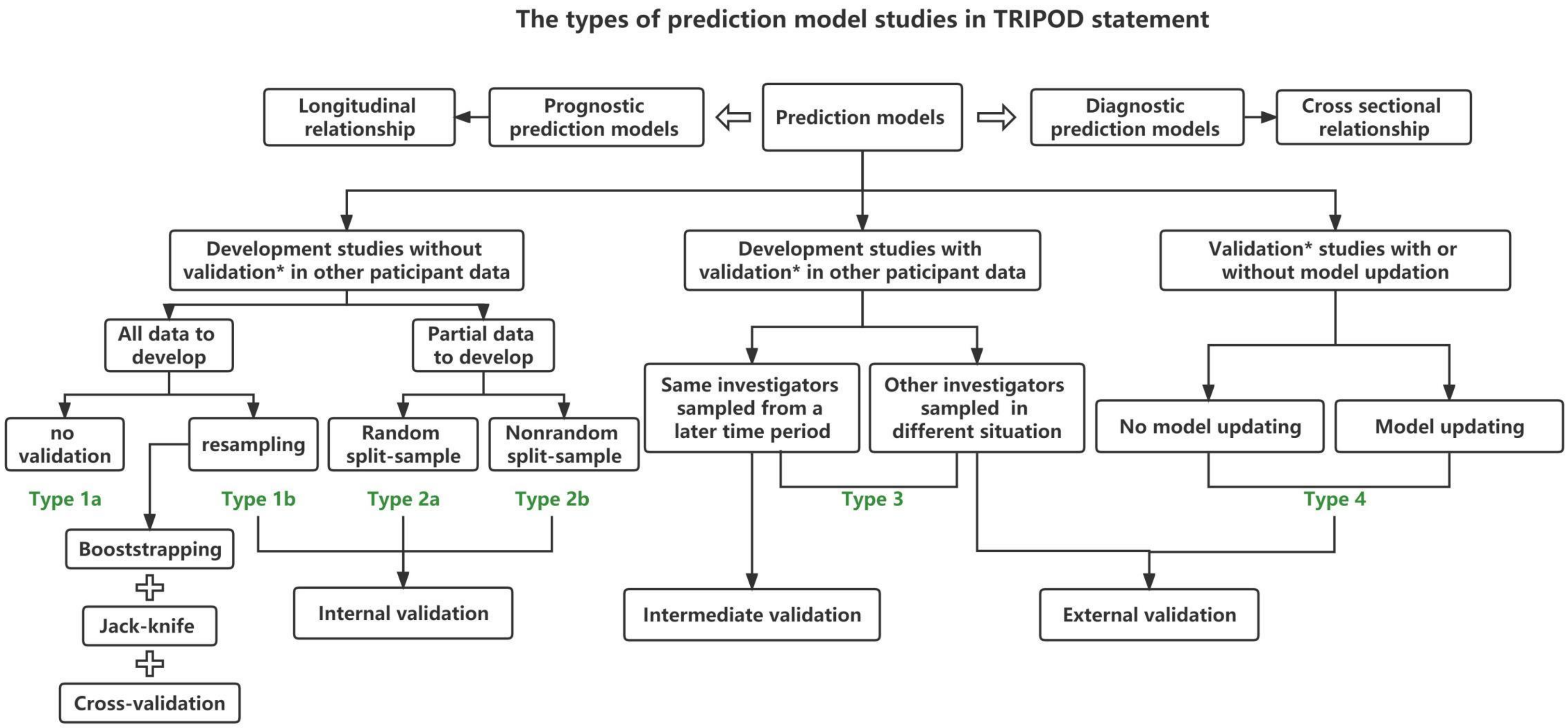

Fig. S1 Types of prediction model studies in TRIPOD statement.

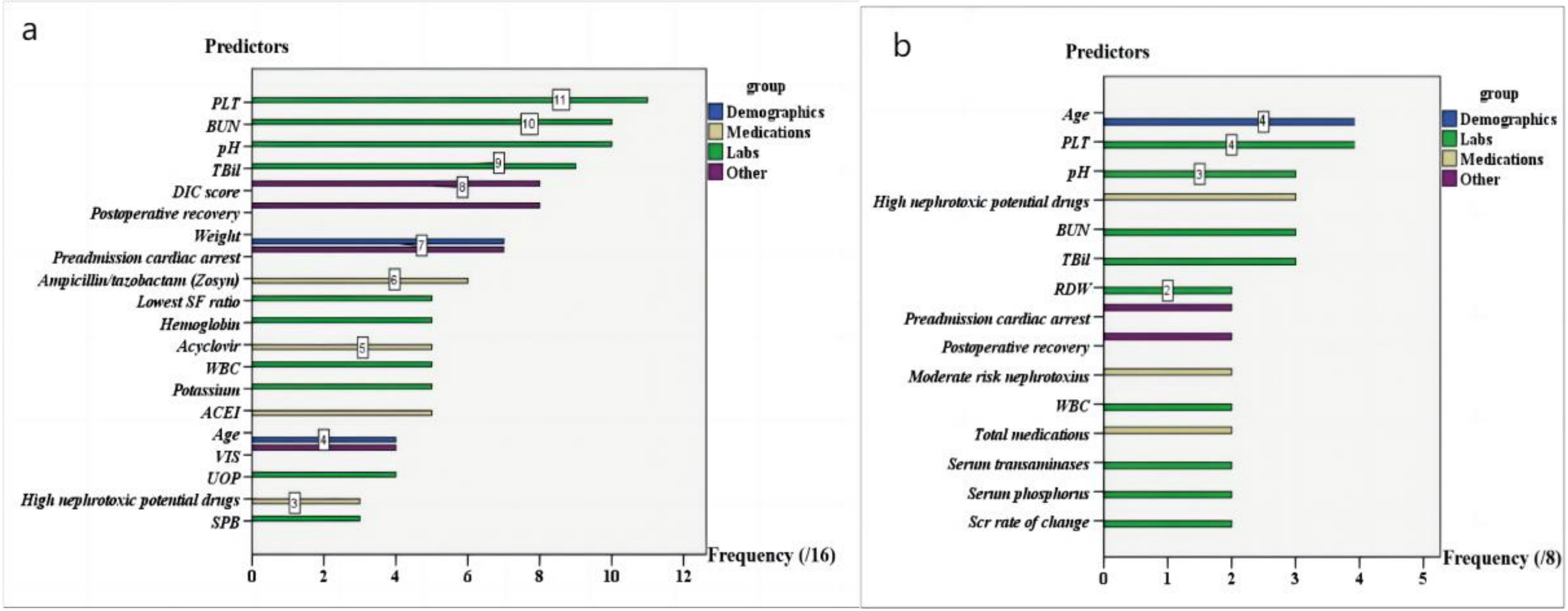

Fig. S2 The most common predictors in prediction models for AKI in hospitalized children. a. Predictors most frequently included in the 16 models; b. Predictors most frequently included in the 8 studies. Labs, laboratory parameters; PLT, platelet count; BUN, blood urea nitrogen; TBil, total bilirubin; DIC score, disseminated intravascular coagulopathy score; Lowest SF ratio, lowest SaO_2/FiO_2 (SF) ratio; WBC, white blood cell count; ACEI, angiotensin-converting enzyme inhibitors; VIS, vasoactive-inotropic score; UOP, urine output; SPB, systolic blood pressure; Postoperative recovery, main reason for admission is post-op observation.
